# Preserved appreciation of aesthetic elements of speech and music prosody in an amusic individual: A holistic approach

**DOI:** 10.1016/j.bandc.2017.03.010

**Published:** 2017-07

**Authors:** Ariadne Loutrari, Marjorie Perlman Lorch

**Affiliations:** Department of Applied Linguistics and Communication, Birkbeck, University of London, UK

**Keywords:** Congenital amusia, Montreal Battery of Evaluation of Amusia, Speech prosody, Music prosody, Auditory perception, Emotional expression

## Abstract

We present a follow-up study on the case of a Greek amusic adult, B.Z., whose impaired performance on scale, contour, interval, and meter was reported by Paraskevopoulos, Tsapkini, and Peretz in 2010, employing a culturally-tailored version of the Montreal Battery of Evaluation of Amusia. In the present study, we administered a novel set of perceptual judgement tasks designed to investigate the ability to appreciate holistic prosodic aspects of ‘expressiveness’ and emotion in phrase length music and speech stimuli. Our results show that, although diagnosed as a congenital amusic, B.Z. scored as well as healthy controls (N = 24) on judging ‘expressiveness’ and emotional prosody in both speech and music stimuli. These findings suggest that the ability to make perceptual judgements about such prosodic qualities may be preserved in individuals who demonstrate difficulties perceiving basic musical features such as melody or rhythm. B.Z.’s case yields new insights into amusia and the processing of speech and music prosody through a holistic approach. The employment of novel stimuli with relatively fewer non-naturalistic manipulations, as developed for this study, may be a useful tool for revealing unexplored aspects of music and speech cognition and offer the possibility to further the investigation of the perception of acoustic streams in more authentic auditory conditions.

## Highlights

• An amusic individual was given novel tasks of speech and music prosody.
• Intact processing of holistic aesthetic aspects of prosody was demonstrated.
• Examination of speech and music prosodic phenomena adds to understanding amusia.

## 1 Introduction

Music processing deficits can arise due to congenital neurogenic anomalies, and are commonly referred to under the term ‘amusia’ (Peretz & Hyde, 2003). Four percent of the population is estimated to manifest this disorder according to a study by Kalmus and Fry (1980). Congenital amusia has been characterised as a lifelong condition in a number of studies (e.g., Hyde et al., 2011, Patel, 2003, Tillmann et al., 2009). This term, however, does not refer to a homogeneous pattern of disorder, as various aspects of music cognition may be differentially affected. Individuals with congenital amusia are found to display difficulties in the perception of melodic and/or rhythmic patterns (Peretz, Champod, & Hyde, 2003). Impaired processing of intonation has also been observed in some (see, for example, Patel, Wong, Foxton, Lochy, & Peretz, 2008).

This heterogeneity is also found in studies investigating the neural underpinnings of congenital amusia. There is evidence of abnormalities in the right secondary auditory cortex (Patel, 2008), right dorsolateral prefrontal cortex (Schaal, Pfeifer, Krause, & Pollok, 2015), and frontotemporal connectivity (Albouy et al., 2013, Hyde et al., 2011, Loui et al., 2009). In general, the neural anomalies giving rise to speech and musical auditory deficits in congenital amusics remain under debate (Chen et al., 2015, Liu et al., 2015).

Current behavioural research on congenital amusia also underscores the heterogeneity of the nature of the impairment, with studies demonstrating a wide variety of processing difficulties in terms of pitch processing and emotion perception, as discussed below. The variation in terms of the manifestations of amusic deficits calls for the use of multiple research designs that can accommodate the intricate and multifaceted nature of the disorder.

### 1.1 Emotional involvement in amusia and links to prosodic appreciation

The complexity of pitch processing deficits in amusia seems to go further beyond the scale, contour, and interval classification. In a meta-analysis of 42 studies involving participants with amusia, Vuvan, Nunes-Silva, and Peretz (2015) found a strong correlation between acoustic pitch processing and musical pitch processing, favouring the interpretation of a general acoustic deficit in amusics. However, closer inspection of individual cases included in their meta-analysis revealed that 9 amusics displayed compromised musical pitch processing with preserved general acoustic processing, while 17 amusics manifested the reverse dissociation.

This variation is not limited to the pitch domain and reveals an even more nuanced picture in the context of musical emotion. McDonald and Stewart (2008) investigated whether music appreciation in amusics is affected by music perception deficits through the use of questionnaires. Overall results showed a low self-reported appreciation for music in comparison to controls. Most amusics reported poor experience of emotional involvement in response to music but some of them shared a profile similar to controls. The existence of subgroups in congenital amusia also appears pronounced in another study of evaluation of music engagement and appreciation (Omigie, Müllensiefen, & Stewart, 2012). The authors employed an experience sampling approach in order to study a number of variables related to listening behaviour and music appreciation in 17 amusic individuals. A relatively large percentage of their amusic participants displayed a profile of music engagement and appreciation that was very close to that of controls. These findings suggest that music appreciation is not necessarily compromised in the presence of congenital amusia and highlight the variability of music deficits in amusics.

Amusics’ processing of musical emotion has been also studied empirically (Ayotte et al., 2002, Gosselin et al., 2015, Marin et al., 2015). What is more relevant to the scope of the present study, however, is to draw attention to the distinction between these ‘labelled’ emotions and those emotions that may contribute to the appreciation of the overall listening experience. This distinction is highlighted by considering the difference between ‘aesthetic’ emotions that are more related to qualities such as admiration for the performer’s skill and ‘everyday’ emotions that are closer to specific emotional labels we use in everyday life (Juslin, 2013). The two aspects appear to be relatively independent of one another. For example, one might be able to successfully judge the mood of a music piece, while, at the same time, being completely unmoved by it (Sloboda, 1991). From this standpoint, we argue that research on emotion in relation to music and speech should be distinguished from the investigation of prosodic appreciation arising from such acoustic streams.

### 1.2 ‘Expressiveness’ in music and speech

Perceptual processing of the aesthetic qualities of musical performance is an important dimension of music cognition that may contribute to further understanding, especially in regard to congenital amusia. Instrumental musical performance, in a similar way to oral spoken performance, combines established rules of tradition with unique elements contributed by the individual performer (Bowen, 1993). These unique qualities of individual performers appear as a result of multiple levels of artistic expertise. Repp (1995) studied expressive timing in piano performance, comparing graduate piano students and experts and found that students were highly consistent in terms of timing strategies, whereas the expert pianists showed great individual variation in deviations from expected timing. Performances rich in expressive features and those that lack these cues are highly contrastive in both music and speech. Being exposed to a computer performance lacking musical microstructure, which includes elements such as temporal deviations, timbre changes, and loudness variation found in human music performance, has a similar effect to being exposed to a stilted delivery of speech (Clynes, 1995). For example, expressive timing in music may be linked to phrase-final lengthening in speech (Palmer & Hutchins, 2006), and the absence of such elements in musical or linguistic phrases constitutes a qualitative difference that comes into play during acoustic processing.

### 1.3 Speech prosody in amusia research and terminology limitations

Speech processing has also been explored in amusia research. Speech comprehension and processing of elements of linguistic prosody in amusics have been discussed in a number of recent studies (see, for example, Hutchins et al., 2010, Jiang et al., 2010, Liu et al., 2015, Liu et al., 2010, Patel et al., 2005, Patel et al., 2008, Stewart, 2011). Emotional prosody in speech processing has also been investigated in amusic individuals (e.g., Lolli et al., 2015, Thompson et al., 2012). However, there are additional aspects of speech prosody that do not necessarily fall under the scope of linguistic and emotional prosody. For instance, the interaction of acoustic cues in the voice of a speaker in various renditions of the same text, as in dramatic performances, can be judged to be different by the listener, although there are not currently detailed enough prosodic labels at the disposal of the scientific community in order to capture what these differences are.

### 1.4 Towards a holistic framework of prosodic phenomena

At the same time, the question arises as to whether these aesthetic prosodic nuances are processed in isolation or holistically. Approaching the complex experience of auditory processing can be viewed through the lens of a holistic approach. This approach is well developed in the domain of vision (see Grossberg & Pinna, 2012) but it could be also fruitful in the auditory domain and, more specifically, in the area of prosody processing. In a recent study, Menninghaus et al. (2015) raise, among other things, the issue of combination of poetic devices in different renditions of spoken proverbs. The combination of these devices, including the prosodic feature of meter, was found to result in different judgements compared to cases where these features were presented in isolation. The authors note that elements of poetic speech depend heavily on context and processing them in combination does not mirror processing each of them in isolation. Audibert, Aubergé, and Rilliard (2005) studied the perception of emotional prosody in a resynthesis-based framework. They found that, although some isolated acoustic features might be more indicative of certain emotional labels, no isolated cue can fully convey all the necessary emotion information conveyed to the listener. This further suggests that as one processes emotional colouring in speech, these cues are not appreciated as features in isolation but are perceived holistically and are associated with a given emotion. The findings of this holistic effect in behavioural studies are supported by a functional magnetic resonance imaging (fMRI) study by Wiethoff et al. (2008). The authors found that processing emotionally intoned words activated neural networks that are different from those seen when investigating individual cues such as pitch, duration, and intensity in isolation, as processing appears to depend on the synergy of such features. As musical performance also displays combinations of the above features, a similar processing paradigm could also apply to the music domain. The above findings are not surprising, as individuals are normally exposed to rich acoustic streams rather than prosodic parameters in isolation. Our approach and research design have been motivated by the above evidence in a parallel effort to employ more naturally-occurring and more fully complex realisations of music and language stimuli.

### 1.5 Background and objectives

We selected a documented amusic individual to test her performance on more holistic aspects of music and language prosody processing. As such, our study represents a follow-up case study on the Greek amusic individual described by Paraskevopoulos, Tsapkini, and Peretz (2010). The authors of the initial study were interested in determining whether the Montreal Battery of Evaluation of Amusia (MBEA; Peretz et al., 2003), which has been widely used in North America and Western Europe, could be employed to diagnose music perception deficits in populations exposed to different musical idioms. They designed a Greek version of the MBEA (GBEA) for populations sharing the Middle-Eastern musical tradition. Results obtained by 30 neurotypical Greek individuals confirmed the importance of using culturally specific materials, as their scores were significantly lower on the MBEA compared to their Canadian counterparts. In contrast, the adapted Greek battery was shown to have a much stronger diagnostic value with smaller standard deviations in the performance of Greek participants across subtests.

In the context of their study, Paraskevopoulos et al. (2010) tested one congenital amusic individual, referred to as B.Z, who was given both the MBEA and the GBEA batteries. They reported that her performance revealed music perception deficits which were more pronounced in the Greek version of the amusia battery. In particular, B.Z.’s performance on the culturally-tailored GBEA tests revealed statistically significant impairments on all pitch subtests: scale task – 19/30; contour task – 17/30; interval task – 16/30. Her rhythm and memory scores on the same battery were within normal limits but her performance on the meter subtest was significantly lower than healthy controls with a score of 15 correct answers out of 30.

The previous study examined the more typically investigated dimensions of pitch, rhythm, and meter in B.Z. In the present follow-up study, we set out to investigate the ability of the same individual to make perceptual judgements of musical and linguistic stimuli with a new range of tasks. With the design of these new tasks we aimed to further explore the relationship between the standard variable dimensions of music processing as tested through tasks included in the existing amusia batteries and the sensitivity to the aesthetic value of music and speech streams which represents an unexplored dimension of cognition in amusic populations.

Thus, the main objective of the current study is to contribute to the understanding of aesthetic appreciation abilities in amusia. Previous music cognition research has often employed computer-manipulated artificial stimuli that are not environmentally valid and can result in examining the auditory abilities of the listener rather than their music cognition (Bigand & Poulin-Charronnat, 2006). We, therefore, generated and used stimuli shifting from strict lab-oriented manipulations so that they could be more similar to naturally-occurring music and speech streams rather than artificial instances of these two domains. We present acoustic conditions in both the music and the speech domain, arguing that the interaction of perceptual features present in our stimuli are interwoven with aesthetic appreciation of such streams. Although specific computerised manipulations are often preferred due to their objectively measured character, they may less directly reflect aspects of human prosody that are not included in existing prosodic labels. Our tasks employ stimuli created in order to explore more implicit quality evaluation of music and speech that is likely to depend on overall properties of the acoustic object.

We call the new percept that we are investigating ‘expressiveness’ and, in the case of music, we define it as an aesthetic attitude towards music that allows discriminations on the basis of ‘music prosody’ as distinct from tune or meter (Loutrari & Lorch, in press). The term ‘music prosody’ has been thoroughly described in Palmer and Hutchins (2006) but, for the purposes of our study, we define it as the sum of the acoustic features that make tunes of identical pitch organisation differ in terms of their aesthetic appeal.

In addition to tasks addressing the perception of ‘expressiveness’, we also included a pair of tasks on basic emotion identification in music and speech as well as a task on speech prosody detection. In all the novel tasks designed for the purposes of this study, we aimed to focus on holistic prosodic perception rather than limiting our attention to a single prosodic aspect at a time. Previous research employing subjective instruments suggests that it is not clear to what extent the traditionally identified amusic deficits can compromise music appreciation. Omigie et al. (2012) did not find a correlation between performance on the MBEA subtests and scores on music engagement in the amusic participants of their study. That is, milder cases of amusia did not correspond to higher level of music engagement and appreciation. We set out to explore whether having a fundamental difficulty in making determinations on micro-level features such as pitch also affects the ability to make holistic judgements on prosodic information.

## 2 Methods

### 2.1 Apparatus

Digital Performer 8 was employed for the generation of all stimuli. Praat software was used to delexicalise linguistic stimuli and a digital piano with weighted keys was used for the recording of the music stimuli. Participants were given the tasks using Windows Media Player and the stimuli were presented binaurally through earphones.

### 2.2 Test materials

Speech and music stimuli had a 44.1 kHz sampling rate and 16-bit resolution.

For the generation of the speech stimuli, naturalistic and intuitive manipulations were made by an adult male Greek native speaker with singing performance experience. As these stimuli were not generated by digital manipulations, spectrograms are presented to demonstrate the differences achieved in terms of acoustic characteristics for the various conditions in particular tasks. To complement the spectrograms, a table providing information on general acoustical characteristics that define the groups of stimuli is presented at the end of this section.

The music stimuli employed in the first task described below were derived from a variation of styles of European Romantic and late-Romantic music including composers such as Frédéric Chopin, Antonín Dvořák, and Sergei Rachmaninoff, relatively well-known in Greece, as well as native Greek composers of the 20th century (Nikos Skalkotas and Yannis Constantinidis). These were included to control for the possibility of an effect of familiarity caused by the repetition of the same music style across the task. Details on this and all other tasks are provided below.

(i) *Expressive music prosody*. This task aimed to investigate sensitivity to elements of music prosody. It included 32 pairs of stimuli with an average duration of 9.3 s. The stimuli were all monophonic (without accompaniment) adaptations of the original melodies. The melodies were executed by the first author on a digital piano with weighted keys and recorded using Digital Performer 8. Tune and meter remained the same across the two stimuli of each matched pair. There were two groups of stimuli, hereafter, ‘expressive’ and ‘non-expressive’. The minimum and maximum durations were 5.24–14.76 s for the ‘expressive’ stimuli and 3.03–13.35 s for the ‘non-expressive’ stimuli.

**Fig. 1:**
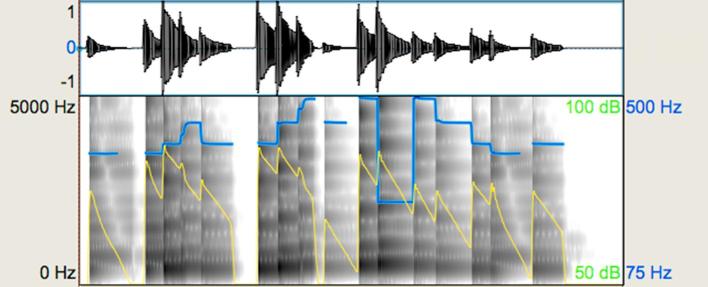
Representative waveform and spectrogram (scale: 0–5000 Hz) for the expressive music prosody task: expressive stimulus. The blue-coloured line shows the pitch contour and the yellow-coloured line shows intensity in the melody. (For interpretation of the references to colour in this figure legend, the reader is referred to the web version of this article.)

**Fig. 2:**
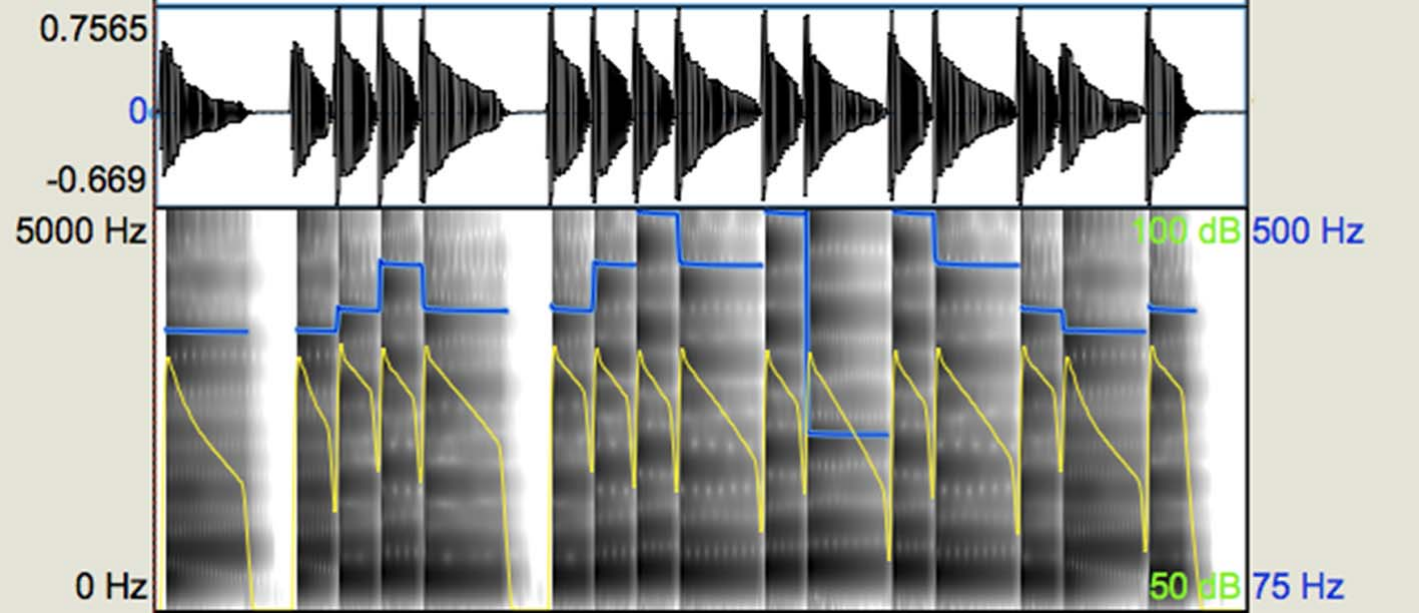
Representative waveform and spectrogram (scale: 0–5000 Hz) for the expressive music prosody task: non-expressive stimulus. The blue-coloured line shows the pitch contour and the yellow-coloured line shows intensity in the melody. (For interpretation of the references to colour in this figure legend, the reader is referred to the web version of this article.)

The ‘expressive’ group displayed deviation from temporal regularity (‘*rubato’*), variation in dynamics, and connected transitions between some notes (legato articulation) within a melody. The ‘non-expressive’ group included melodies with temporal regularity, equal loudness across notes, and heavily accented notes with relatively shortened duration (referred to as ‘*marcato*’ articulation in musical notation). Using Digital Performer 8, the exact value of every note and rest was assigned and loudness variation was removed. The ‘*marcato*’ articulation was also technically generated on Digital Performer 8 to ensure that there would not be variations in articulation. Fig. 1, Fig. 2 show waveforms and spectrograms for the ‘expressive’ and the ‘non-expressive’ version of a representative melody. As can be seen in these acoustic representations, although the pitch sequence is identical across stimuli, the pitch contour of the spectrograms displays slight differences that reflect the difference in articulation. More specifically, it can be seen that the line endings in the ‘expressive’ spectrogram are less perpendicular compared to the ‘non-expressive’. In addition, the dynamics in the ‘expressive’ stimulus vary in contrast to the ‘non-expressive’ one where this loudness variation is not present. Part of the variation in the dynamics of the expressive stimuli is the result of the differences in articulation of one or more notes. Deviation from temporal regularity can be appreciated in the waveforms. The ‘expressive’ stimulus violates the assigned duration of notes for the sake of expressive phrasing, whereas the notes of the ‘non-expressive’ stimulus have the exact duration provided in the scores. These differences are also reflected in the mean duration difference between expressive and non-expressive stimuli (see Table 2).

Half of the pairs included identical stimuli and half of them consisted of pairs of stimuli comprised of one expressive and one non-expressive rendition of the same melody.

(ii) *Expressive speech prosody*. This task was designed to assess perception of prosodic inflection in speech with preserved lexical content. It included 32 pairs of semantically neutral utterances spoken by a male native-Greek speaker. The utterances had an average duration of 8.35 s. Minimum and maximum durations for the expressive stimuli were 6.44–11.93 s and 6.08–9.88 s for the non-expressive ones. The average duration for the expressive stimuli versus the non-expressive ones was again slightly longer as in the music task (Table 2). As also shown in Table 2, stimuli tended to be louder on average in the non-expressive condition and had smaller standard deviations from the average F_0_. The utterances were spoken in two conditions: natural prosodic inflection and minimum possible variation of prosodic features.

For the prosodically marked stimuli, the speaker was instructed to employ pitch, loudness, and duration variation in his speech and also to pause between tone units. For the prosodically unmarked ones, the speaker was instructed to imitate synthetic speech by limiting variation of pitch, loudness, and pausing as much as possible. These pairs of stimuli were designed to create stimuli that were more or less expressive or aesthetically interesting for the listener.

**Fig. 3:**
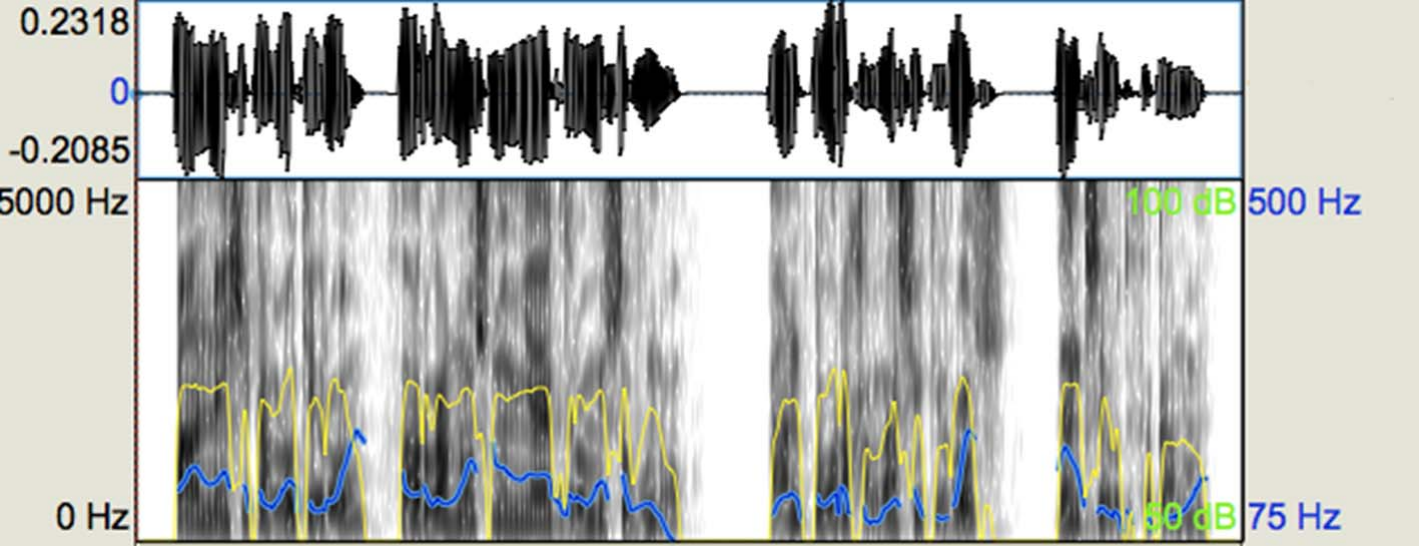
Representative waveform and spectrogram (scale: 0–5000 Hz) for the expressive speech prosody task: expressive stimulus. The blue-coloured line shows the pitch contour and the yellow-coloured line shows the intensity of the utterance. (For interpretation of the references to colour in this figure legend, the reader is referred to the web version of this article.)

**Fig. 4:**
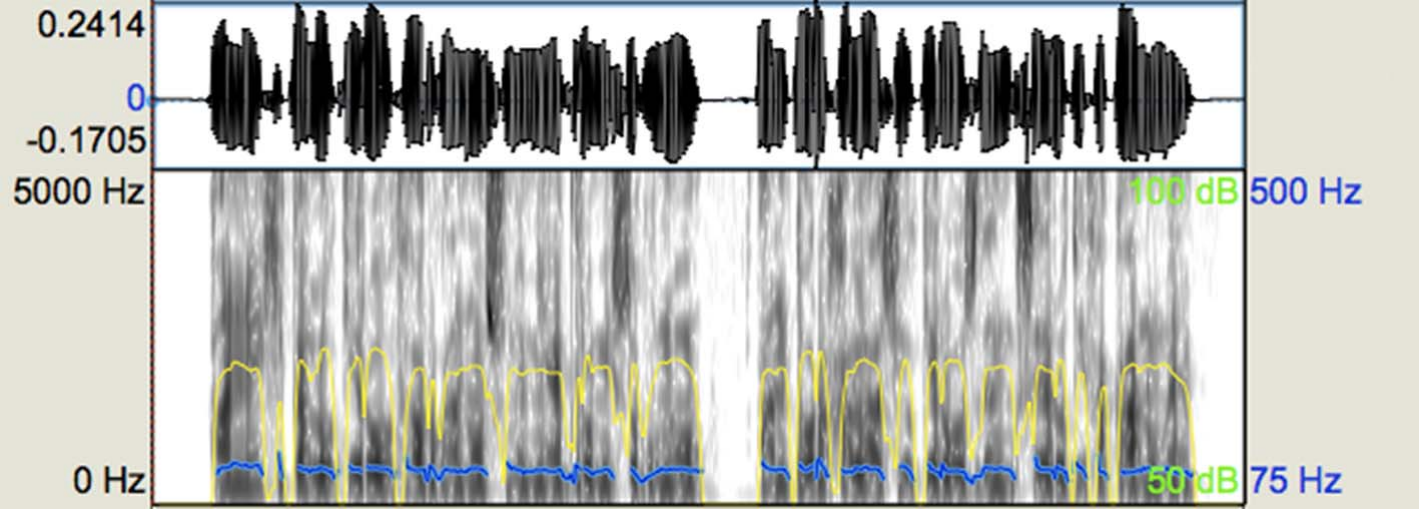
Representative waveform, spectrogram (scale: 0–5000 Hz) for the expressive speech prosody task: non-expressive stimulus. The blue-coloured line shows the pitch contour and the yellow-coloured line shows the intensity of the utterance. (For interpretation of the references to colour in this figure legend, the reader is referred to the web version of this article.)

**Fig. 5:**
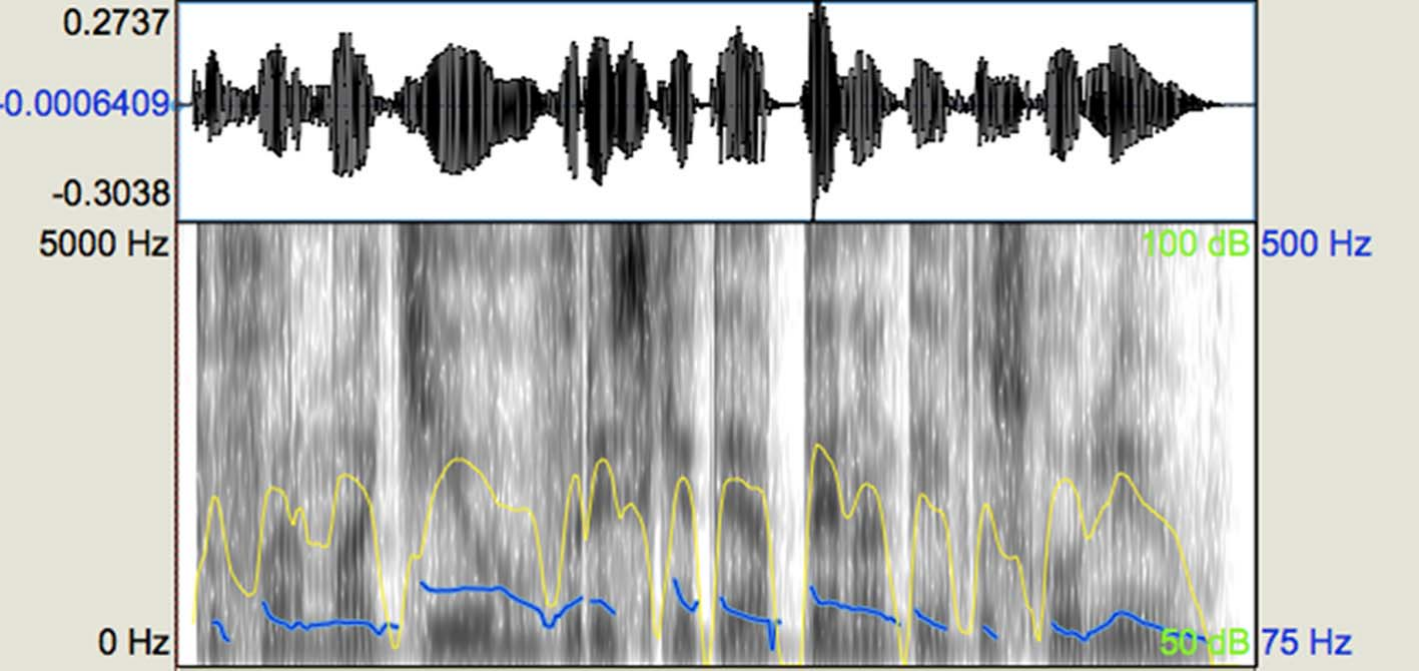
Representative waveform and spectrogram (scale: 0–5000 Hz) for the emotional speech prosody task: sad stimulus. The blue-coloured line shows the pitch contour and the yellow-coloured line shows the intensity of the utterance. (For interpretation of the references to colour in this figure legend, the reader is referred to the web version of this article.)

**Fig. 6:**
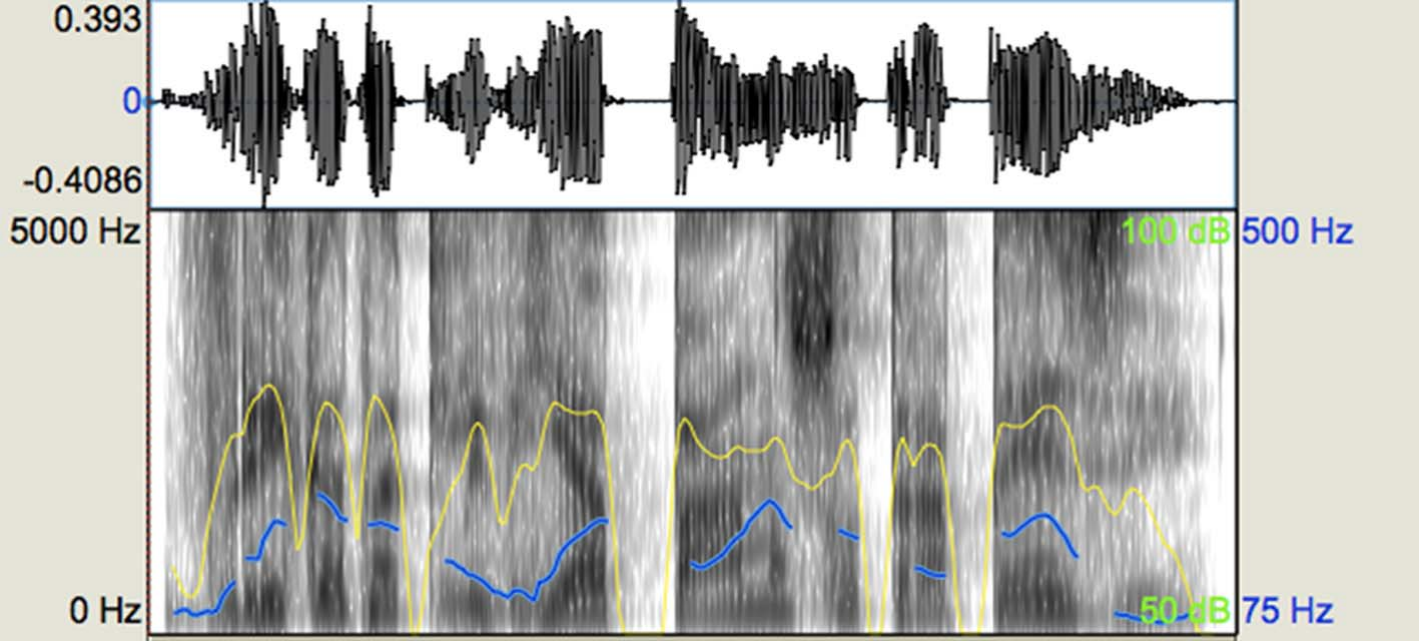
Representative waveform and spectrogram (scale: 0–5000 Hz) for the emotional speech prosody task: happy stimulus. The blue-coloured line shows the pitch contour and the yellow-coloured line shows the intensity of the utterance. (For interpretation of the references to colour in this figure legend, the reader is referred to the web version of this article.)

In Fig. 3, Fig. 4, the reader can appreciate visually the differences between the stimuli of a representative pair intending to evoke a ‘different’ judgement. Pitch contours produced by the speaker (as indicated by the blue line), while not totally flat, had very limited variation in the ‘non-expressive’ stimulus condition as compared to its ‘expressive’ counterpart. There is also a slight difference in dynamics, which is more evident in the second part of both spectrograms indicated by the yellow lines. In addition, more pauses are present in the ‘expressive’ utterance resulting in 4 breath groups versus 2 breath groups of the ‘non-expressive’ utterance, as shown in the waveforms. It can be also appreciated that differences in the duration patterns of the two stimuli provide an additional differentiating cue.

(iii) *Speech prosody detection*. This task assessed ability to detect speech in a delexicalised context. It included 64 stimuli that had prosodic inflection or displayed more limited variation in terms of pitch loudness, pausing, and duration patterns. The utterances had an average duration of 8.35 s. The stimuli employed in this task were the same as those used in the expressive speech prosody task, but Praat software was used to remove frequencies above 300 Hz from all stimuli with low-pass filtering. The resulting speech was semantically unintelligible but prosodic cues were preserved. Stimuli intending to evoke a ‘non-speech’ judgement were the low-pass filtered versions of the stimuli that imitated synthetic speech. Those that intended to evoke a ‘speech’ judgement were the low-pass filtered versions of the parent stimuli in the expressive speech prosody task bearing prosodic variation. Participants were blind to the fact that all stimuli were originally derived from speech.

(iv) *Emotional speech prosody*. This task was designed to assess basic emotional prosody perception in semantically neutral utterances. This was an identification judgement involving the two emotions ‘happiness’ and ‘sadness’. The investigation of this basic binary judgement of emotional speech prosody was included in order to compare participants’ performance on this task to other perceptual judgements of stimuli related to aspects of ‘expressiveness’. It included 32 stimuli in total with an average utterance duration of 2.51 s. ‘Happy’ stimuli had a minimum-maximum duration of 1.08–2.64 s and ‘sad’ stimuli had a minimum-maximum duration of 1.83–3.87 s. The stimuli were spoken by the Greek male native speaker in either happy or sad emotional tones. Fig. 5, Fig. 6 show representative waveforms and spectrograms for this task. As would be expected, the ‘happy’ stimulus has higher pitch and larger intervals relative to the ‘sad’ stimulus. Duration patterns and loudness also display differences. Note that this task did not involve comparisons and, therefore, the stimuli here correspond to completely different utterances. Overall, the average duration of ‘happy’ stimuli was shorter, the average intensity relatively higher, and the average F_0_ much higher compared to the ‘sad’ stimuli (Table 2).

**Fig. 7:**
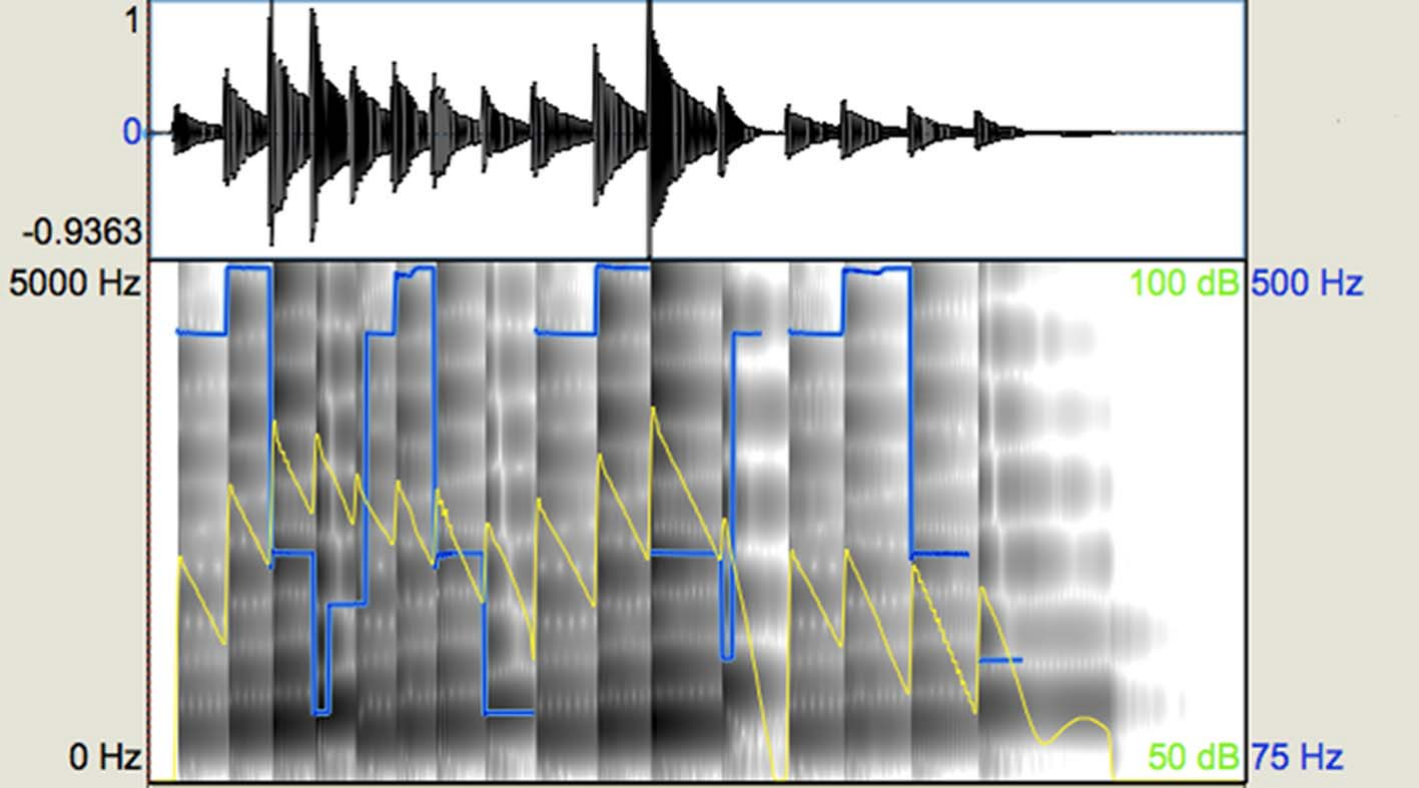
Representative waveform and spectrogram (scale: 0–5000 Hz) for the emotional music prosody task: sad melody. The blue-coloured line shows the pitch contour and the yellow-coloured line shows the intensity of the melody. (For interpretation of the references to colour in this figure legend, the reader is referred to the web version of this article.)

**Fig. 8:**
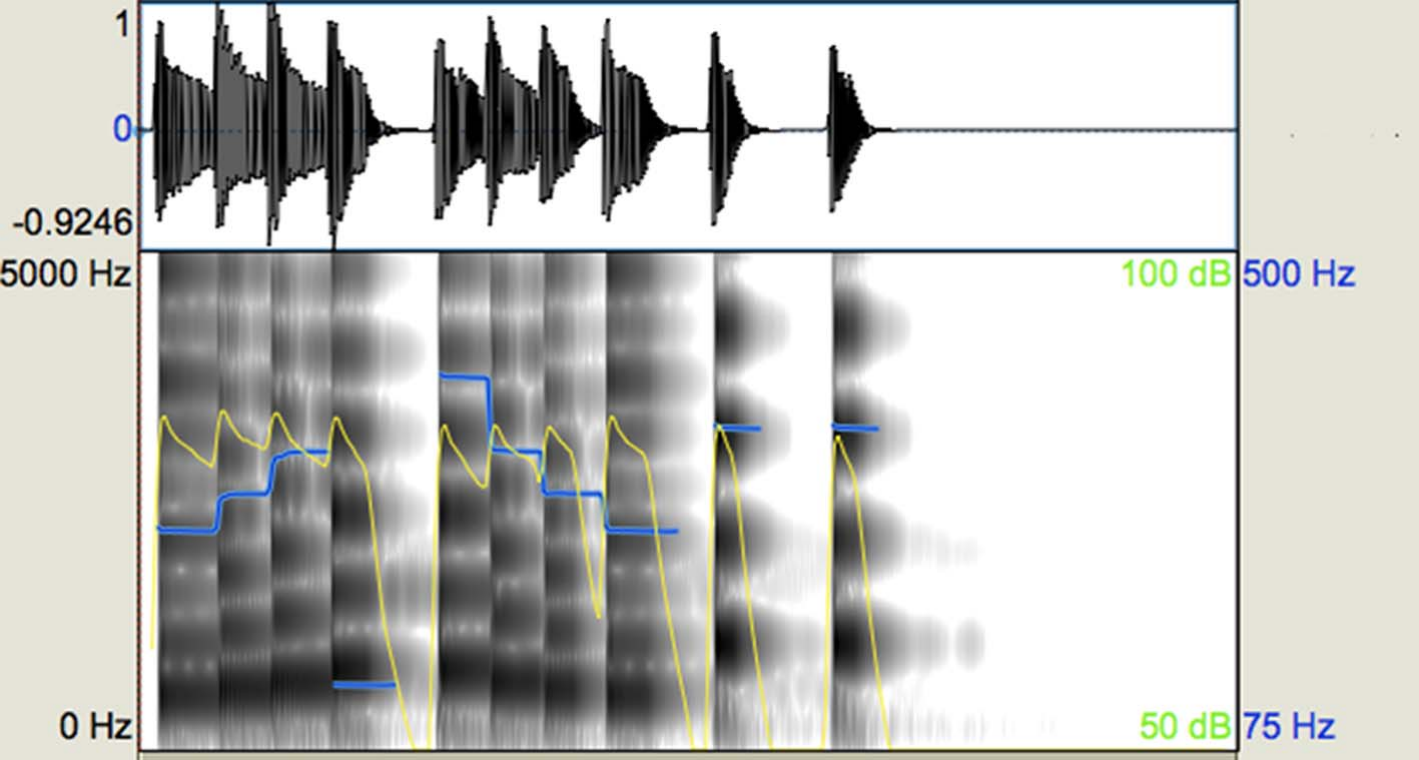
Representative waveform and spectrogram (scale: 0–5000 Hz) for the emotional music prosody task: happy melody. The blue-coloured line shows the pitch contour and the yellow-coloured line shows the intensity of the melody. (For interpretation of the references to colour in this figure legend, the reader is referred to the web version of this article.)

(v) *Emotional music prosody*. The purpose of this task was to explore perception of emotional character in music with two contrastive emotions; happiness and sadness. It comprised of 32 novel melodies in total of an average duration of 6.39 s. Minimum-maximum durations for the ‘happy’ stimuli were 3.18–7.17 s and 6.38–11.99 s for the ‘sad’ stimuli. These were composed and played by the first author on a digital piano with weighted keys and recorded on Digital Performer 8. Melodies intending to evoke a ‘happy’ identification were composed in major mode, had a fast tempo, high loudness, and often quick staccato motifs. Those intending to evoke a ‘sad’ identification were composed in minor mode, had a slower tempo, lower loudness, and the articulation between notes was usually legato. The reader can visually appreciate some of these acoustic differences in quality in Fig. 7, Fig. 8. In terms of overall contrasts between the stimuli pairs, the ‘sad’ melodies were much longer and relatively less loud than the ‘happy’ melodies (see also Table 2).

Table 1 presents a summary of the number of stimuli for each task, the response mode as well as the probability of correctly guessing the answer.

**Table 1:** Variables for number of stimuli, response mode, and guessing probability of the tasks used in the study.

**Tasks**	**Expressive music prosody**	**Expressive speech prosody**	**Speech prosody detection**	**Emotional speech prosody**	**Emotional music prosody**
Stimuli	64	64	64	32	32
Response mode	Same-different judgement	Same-different judgement	4 options, forced choice	Identification	Identification
Guessing probability	50%	50%	25%	50%	50%

**Table 2:** General acoustical features across groups of stimuli for 4 tasks. The speech prosody detection task is not included, as it is a low-pass filtered version of the expressive speech prosody task.

Acoustical features	Mean duration (sec)	Duration SD	Mean intensity (dB)	Intensity SD	Mean F_0_ (Hz)	F_0_ SD
*Expressive music prosody*						
Expressive	9.99	2.69	59.35	8.99	N/A^a^	N/A
Non-expressive	8.62	3.17	60.38	16.32	N/A	N/A

*Expressive speech prosody*						
Expressive	8.60	1.31	42.27	7.77	137.49	11.58
Non-expressive	8.10	1.02	56.70	2.77	121.18	4.93

*Emotional speech prosody*						
Happy	2.20	0.41	66.80	1.73	180.75	17.67
Sad	2.82	0.54	59.70	2.54	116	8.51

*Emotional music prosody*						
Happy	4.40	0.94	62.59	3.48	213.43	18.20
Sad	8.38	1.82	54.78	4.97	228.98	20.63

a Expressive and non-expressive stimuli of the expressive music prosody task had identical pitch organisation and, therefore, pitch-related information is not reported in this table for this task.

Table 2 presents a summary of the acoustic characteristics of the different groups of stimuli. Note that in the expressive music prosody task, the mean and standard deviation for the intensity of non-expressive stimuli was identical across the notes of each stimulus. However, the figures included in the table refer to the mean intensity across all notes of all stimuli in the non-expressive condition.

### 2.3 Procedure

In the previous section, the tasks were described in an order that reflects the methodological rationale for the sake of clarity. However, the order of task presentation to participants was as follows: speech prosody detection, expressive music prosody, expressive speech prosody, emotional speech prosody, and emotional music prosody.

When testing participants, the speech prosody detection task was the first to be presented due to the results we obtained from an earlier piloting phase. In the pilot study, the expressive speech prosody task was presented before the speech prosody detection task. However, pilot participants’ comments during the debriefing indicated that there were unwanted task presentation order effects. Participants appeared to be primed to recognise the delexicalised speech stimuli in the speech prosody detection task when they were first exposed to the stimuli with preserved lexical information in the expressive speech prosody task. This outcome in the pilot study motivated a change in presentation order. It is noted that in the debriefing after the test phase with the present control participants, no individual reported having noticed any similarity between the stimuli used in the speech prosody detection task and the expressive speech prosody task. The speech prosody detection task allowed for the participants to choose one of four possible answers: the first stimulus is speech, the second stimulus is speech, both stimuli are speech, or neither of the stimuli is speech.

The response mode of the expressive music prosody task required same/different discrimination judgements. Participants were instructed to base their judgement on whether the performer was equally expressive across the two melodies of each question.

Same/different discrimination judgements were also required for the expressive speech prosody task. Participants were informed that they would be asked to listen to an actor saying his lines and judge whether the pairs of stimuli were different or equally expressive. It was made clear to them that the words included in the utterances of each pair would be always the same and that they should choose the answer ‘different’ if they detected small differences in how the speaker sounded.

For the emotional speech prosody task, individuals were required to respond to each stimulus, judging them as either conveying happy or sad emotion. Participants had to identify the intended emotion instead of providing comparison judgements. They were instructed to only attend to the tone of the speaker and not its lexical content.

Similar to the emotional speech prosody task, in the emotional music prosody task, the listener had to identify the intended emotion as being either happy or sad.

#### 2.3.1 Ethics

Ethics approval was granted according to the procedures of the Ethics Committee of Birkbeck, University of London. The study was deemed to entail minimal risk for participants and limited potential to cause distress. The researchers protected the physical, social, and psychosocial wellbeing of those participating in the study as well as their confidentiality. Consent was obtained from all participants after informing them of what was entailed in participating in the study and their right to withdraw. All participants were treated in accordance with The Code of Ethics of the World Medical Association (Declaration of Helsinki).

#### 2.3.2 Case: B.Z.

As described by Paraskevopoulos et al. (2010), B.Z. is a congenitally amusic Greek-speaking monolingual individual with tertiary education and professional degrees (MD and PhD in Medical Sciences). She reported not being able to remember tunes or dance to music but not experiencing any other difficulties. At the time, B.Z. was tested on 16 neuropsychological tests covering working memory, recall, visual memory, attention, word fluency, and executive functions and was found to perform within normal limits in all sections of neuropsychological testing without demonstrating any age-related impairments. She was 63 years old at the time she was tested on the MBEA and the GBEA and 67 when she was tested on our tasks. All tasks were given in a quiet room in her house.

#### 2.3.3 Neurotypical individuals

Twenty-four healthy participants were recruited through the music communities and through word of mouth in and around Thessaloniki and Chios, Greece to take part in this study and serve as controls for the novel tasks. They were all right-handed, monolingual Greek speakers with a mean age of 50.29 years (range 45–60 years, S.D. = 5.16). As previous studies had reported the effect of musical experience on the performance on music and/or speech tasks (e.g., Besson et al., 2007, Marques et al., 2007, Schön et al., 2004), approximately half of the group (n = 14) were selected for having previous musical experience, as defined by 3 years (mean = 2.92, S.D. = 0.61) of playing a musical instrument or singing in a choir, while the others (n = 10) had no music experience. All were tested in a quiet room in their house. The controls’ performance was taken as a general baseline determination of task performance and is employed here as a point of reference to compare neurotypical to amusic performance.

## 3 Results

Although there was some heterogeneity in the performance of the group of neurotypical control participants overall, no group differences were found with respect to the musical experience variable on any of the tasks. Independent t-tests were carried out on the performance of individuals with music experience (n = 14) and others that were musically naïve (n = 10). No statistically significant differences were found on any of the 5 tasks: ‘expressive music prosody’ t(22) = −0.17, p = 0.87, ‘expressive speech prosody’ t(22) = −0.09, p = 0.92, ‘speech prosody detection’ t(22) = 1.3, p = 0.18, ‘emotional speech prosody’ t(22) = −0.87, p = 0.39, ‘emotional music prosody’ t(22) = −0.05, p = 0.95. These results show that music experience ($\overset{¯}{\text{x}}$ = 3 years) did not differentiate the performance of these individuals in relation to their musically uneducated counterparts. Hence, the neurotypical individuals were treated as one group (n = 24).

B.Z.’s performance was within the range of the neurotypical participants’ performance on all the tasks, was relatively higher than the mean of neurotypical participants on most tasks, and achieved ceiling performance on one task. The Crawford and Garthwaite (2002) modified *t*-test was used in order to determine whether B.Z.’s scores were statistically significantly different from the neurotypical participants. No significant difference was found when comparing the amusic individual’s performance with the neurotypical control group.

Moreover, the results of the Crawford and Garthwaite (2002) modified *t-*test indicate that a large percentage of the normal population is estimated to fall below her performance on a number of tasks: (1) the expressive speech prosody task (70.70%); (2) the speech prosody detection task (69.46%); and (3) the emotional speech prosody task (65.77%). The percentage of the neurotypical population whose performance is estimated to fall below B.Z.’s scores is also considerable in the expressive music prosody task and the emotional music prosody task, 48.04% and 24.12% respectively. Details from the statistical test and the performance of controls (mean, standard deviation, minimum and maximum achieved scores) can be found in Table 3.

**Table 3:** B.Z.'s performance on all tasks. Scores are reported in relation to the neurotypical participants using Crawford and Garthwaite’s (2002) modified *t*-test. Percentile score refers to the estimated percentage of normal population falling below B.Z.'s score. The maximum achievable score was 32 for all tasks.

Tasks performance items (n = 32)	B.Z. score	Controls (n = 24) mean score	Controls S.D.	Controls score min-max	*t*-value	2-tailed *p* value	Percentile score (%)
Expressive music prosody	24	24.16	3.15	17–29	−0.050	0.961	48.04
Expressive speech prosody	31	30.16	1.49	25–32	−0.552	0.586	70.70
Speech prosody detection	31	28.83	4.12	19–32	0.516	0.611	69.46
Emotional speech prosody	32	31.79	0.50	30–32	0.412	0.685	65.77
Emotional music prosody	29	30.37	1.88	27–32	−0.714	0.482	24.12

## 4 Discussion

### 4.1 ‘Expressiveness’ as a new variable in amusia research

With respect to B.Z.’s previously demonstrated performance on the GBEA, Paraskevopoulos et al. (2010) reported that she scored within normal limits only on the rhythm subtest and the memory subtest. On the rest of the tests, her performance was significantly poorer than controls: scale, t(29) = −2.58, p = 0.015, contour, t(29) = −2.46, p = 0.02, interval, t(29) = −3.53, p = 0.001, and meter, t(29) = −3.078, p = 0.005 (Paraskevopoulos et al., 2010). Taken together with B.Z.’s good performance on the novel tasks investigating aspects of ‘expressiveness’ presented here, the data suggest a potential independence of scale, contour, interval, and meter judgements from judgements of aesthetic appreciation and emotion. More specifically, given B.Z.’s compromised performance on the GBEA contour subtest, it is notable that she performed very well on the expressive speech prosody task and the speech prosody detection task where pitch was one of the main differentiating features between stimuli that were to be classified as ‘different’. This might suggest a difference between pitch processing as assessed in the current amusia batteries and processing of pitch in speech in the presence of additional prosodic cues. Her performance on the GBEA stands in sharp contrast with her near-ceiling performance on both the expressive speech prosody task and the speech prosody detection task. As our tasks did not address pitch processing in isolation, we are not in a position to attribute her performance to a music/speech dissociation. However, we can safely argue that her ability to differentiate between utterances of different degrees of prosodic inflection is intact.

In the expressive music prosody task, pitch was not a differentiating factor, as all pairs of stimuli had identical contours and two scenarios are possible. One interpretation of the current results is that B.Z.’s spared rhythm perception, as shown in both the MBEA and the GBEA, contributed to her ability to discriminate between different stimuli. This raises the question of whether the ability to perceive deviation from temporal regularity (‘*rubato*’) is directly related to the ability to perform rhythm discriminations as those included in the amusia batteries where different stimuli are yielded by modifying the duration of only two adjacent notes. The expressive music prosody task does not demand attention to every single note and adaptations of this task in future studies could shed some light on the above question. The second scenario favours the interpretation that perception of holistic prosodic qualities in the melodies accounts for B.Z.’s normal performance. That is, the interaction of deviation from temporal regularity, variation in dynamics, and differences in articulation might have holistically facilitated her judgements. We are grateful to the anonymous reviewer who suggested that it is possible to frame the account of B.Z’s performance in terms of the detection of some micro-changes or differences on a local level rather than through reference to the appreciation of holistic properties. This helpfully highlights the tension between a componential approach and a more phenomenological orientation to higher order perceptual behaviour. We offer this interpretation framed within the latter perspective as one that may hold potential for new ways of investigating such auditory experiences.

Recent studies of the performance of amusic individuals on different aspects of acoustic processing have presented a heterogeneous and complex picture. The good performance of B.Z. on these novel judgments of music and speech prosody, while having been previously documented to have difficulty with aspects of pitch and meter, raises further questions about what processes are compromised in individuals with congenital amusia. Historically, the condition has been also characterised by the terms ‘tone-deafness’, ‘dysmusia’, and ‘dysmelodia’ (Peretz & Hyde, 2003). Presently, the determination of amusia is typically based on a difficulty in perception of pitch, rhythm, and music memory. However, the results of the present study suggest that investigating the perception of ‘expressiveness’ in music stimuli may capture additional relevant components of music cognition. Our data reveal that some aspects of music cognition are preserved in this individual. Further determination of the underlying properties of such auditory perception abilities in the amusic population is warranted. We suggest that ability to appreciate music does not exclusively depend on intact pitch perception. The results of the present study give an indication that this perceptual ability, as tested through the expressive music prosody task, can be preserved in the presence of congenital amusia. The ability to make such judgements may reflect some preserved aspect of appreciation of musical stimuli. However, it must be acknowledged that the ability to make such auditory discriminations is not sufficient to ensure genuine enjoyment of music in everyday life.

This type of engagement as well as the type of processing required in our expressive speech prosody task is likely to include a different neural network that operates regardless of the neurogenic anomalies present in congenital amusia. A reduction of white matter concentration in the right inferior frontal gyrus of amusic individuals might account for the performance of amusic individuals in pitch-based musical tasks (Hyde, Zatorre, Griffiths, Lerch, & Peretz, 2006). The right inferior frontal gyrus in amusics has been also shown to have increased grey matter concentration (Hyde et al., 2007). Research with healthy participants indicates that this area subserves pitch comparisons which require the individual to actively maintain pitch information (Zatorre, Evans, & Meyer, 1994). Behavioural data stemming from studies with amusic participants are in line with this evidence (see, for example, Tillmann et al., 2009).

The above findings suggest that when an amusic participant is tested on tasks that require close attention to make comparisons of pitch patterns, white and grey matter anomalies in their prefrontal cortex may compromise their pitch-related working memory. This type of judgement is the key to successful performance on the pitch subtests of the MBEA and the GBEA. As mentioned above, we do not know if B.Z.’s ability to perceive features such as deviation from temporal regularity as in our expressive music prosody task is related to her preserved ability to perceive rhythm differences tested through the MBEA and the GBEA where manipulations are applied only to two adjacent notes. With respect to her scores on the pitch subtests of the GBEA, it can be argued that inability to perform short-term memory judgements does not entail inability to engage in other types of pitch processing such as these tested through the expressive speech prosody task where pitch differences were present over longer chunks. It can be further argued that processing of other types of acoustic cues which might be impaired in congenital amusia does not necessarily compromise judgements based on holistic processing of the stimulus. We propose that holistic processing in amusics is not related to the executive function difficulties that they experience when required to pay close attention in order to catch isolated manipulations in a pair of stimuli.

The above interpretation may not, however, hold for some subgroups of amusic individuals who represent different types of neurofunctional architecture, as pointed out in the introduction. Based on the premise that amusia is a heterogeneous disorder, the above proposition can hold true for the amusics whose impaired performance stems from their inability to maintain pitch information in order to successfully compare a pair of melodies. Further work will be needed in order to test the above hypothesis through both behavioural and functional brain imaging studies.

Intact holistic processing can enable perception of different degrees of prosodic inflection in speech in the presence of poor performance on judgments related to isolated acoustic features. In addition, music appreciation should not be necessarily thought of as solely relying on pitch perception, as appreciation can be possible in the presence of a pitch processing deficit. Although the relationship of amusia with ‘everyday emotions’ has been the focus of other studies (as dealt with in the following section), evidence on the perception of ‘aesthetic emotions’ in amusic individuals has not been reported in previous literature.

### 4.2 Complexities in evaluating emotion perception in amusia

There is some evidence suggesting that perception of musical emotion can be preserved in the presence of amusia. Processing of happy and sad music stimuli has been shown to be spared in amusics (Ayotte et al., 2002). In a more recent study, Gosselin et al. (2015) showed that amusics have normal recognition of happy, sad, scary, and peaceful emotions in music and they attributed their access to emotional cues, mainly to pulse clarity and timbre, but also large mean pitch differences in the case of happy-sad judgements. Marin et al. (2015) studied amusics’ sensitivity to emotional colouring through the use of major and minor chords in both a sine-tone and a complex-tone version. They found that amusics were sensitive to dissonance and consonance, although this was more pronounced in the case of complex tones. Amusics were also found to perceive major triads as happy and minor triads as sad in the complex-tone condition.

However, evaluation of emotion perception is not simple to carry out and impairments may remain hidden due to methodological choices. For example, judgements might be more based on the presence of contrastive cues among the tested emotions rather than on intact perception of these emotions. A patient with apperceptive music agnosia subsequent to a right temporo-parietal lesion, J.M., reported in Baird, Walker, Biggs, and Robinson (2014) was found to have impaired recognition of sad and peaceful emotions in music but preserved recognition of happy and scary emotions. Results from J.M.’s performance on the MBEA show average performance on the meter subtest and compromised performance on all the other subtests. Baird et al. (2014) suggested that preserved perception of meter can be sufficient for the identification of happy and scary emotions but not of sad and peaceful. This conclusion further suggests that the acoustic cues that are more prominent in the acoustic stimuli can affect the performance of participants. Moreover, the combination of the selected emotions can be critical to the results. B.Z. was found to have impaired performance on the GBEA meter subtest while she was able to recognise happiness versus sadness in speech and music. Differences in mean duration of stimuli (happy versus sad) in the emotional speech prosody task, which were even more pronounced in the emotional music prosody task, might have facilitated her judgements. Across these tasks, differences in mean intensity and mean F_0_ might have also contributed to her successful performance. However, it is possible that the inclusion of additional and less contrastive emotions might have presented a more complex picture.

The challenges of studying emotion perception are also present in speech. The range of emotions that are studied is again a factor that comes into play. For example, in Thompson et al. (2012), amusics were found to have impaired perception of speech prosody carrying emotions of happiness, tenderness, sadness, and irritation. If such a range is narrowed down to fewer and more contrasting emotions, such as happiness and sadness, as in the present study, performance might not reflect the same trends. In our study, B.Z. was only required to identify happy and sad speech prosody and she scored at ceiling. It should be noted that our aim was not to provide an exhaustive assessment of B.Z.’s emotion perception abilities but rather to evaluate whether basic emotion perception was preserved in her performance.

### 4.3 Prosody and amusia

We chose to look at the perception of holistic prosodic qualities in B.Z., shifting away from the study of isolated components of prosodic patterns. B.Z. scored above the mean of the neurotypical group on three tasks: the speech prosody detection, the expressive speech prosody, and the emotional speech prosody. In contrast to previous studies examining perception of sentences of indicative versus interrogative mood, sentences displaying differences on the words bearing emphasis, or sentences differing uniquely in terms of grouping, B.Z. was presented with stimuli that encompassed a combination of prosodic features. She was demonstrated to be successful in discriminating between utterances with richer prosodic inflection in comparison to others with limited prosodic variation. She was also able to identify speech in a delexicalised context, which indicated that she was able to base her judgments solely on prosodic inflection.

The speech detection task provided an implicit method to test perception of speech contours. Participants were required to indicate whether the stimuli they were exposed to corresponded to speech or some kind of noise. Part of the rationale of this task was similar to that of Liu, Jiang et al. (2015) who examined speech perception in amusia in everyday listening situations. Although our task did not address speech intelligibility, it explored an everyday ability; whether one is able to detect speech behind closed doors as opposed to perceiving it as irrelevant noise. B.Z.’s good performance in identifying normal low-pass filtered speech contours versus low-pass filtered utterances with slight prosodic variation reveals an underlying preserved ability to identify natural instances of speech despite the absence of lexical content.

On a general level, it has been argued that the emotional response to music can include experience of consonance/dissonance, recognition of emotions, the transformative experience of chills, and the motivation to make music as part of one’s life (Stewart, 2011). We would like to add aesthetic emotions as part of a prosodic aspect of engagement. The development of the concept of ‘expressiveness’ is intended as a key step towards the broader understanding of prosodic components of music and speech cognition, underscoring the rich phenomenological experience of appreciation of the sound stream (Loutrari and Lorch, in press).

Additional empirical research is warranted in order to further test the relative contribution of a dynamic interaction of aesthetic prosodic features in addition to the processing of acoustic features in isolation. We suggest that the domain of auditory processing may benefit from a more phenomenological approach similar to that employed in vision perception, thus adding to the componential approach that focuses on micro-level features that is widely used in the field. This approach may be the source of additional insights into disorders of musical listening and expand on our understanding of more holistic aspects of music processing that might remain underappreciated when deficits are tested through the manipulation of isolated acoustic cues.

## 5 Conclusions

The present study demonstrated preserved processing of speech and music prosody in an individual with congenital amusia with tasks designed to study more authentic auditory conditions. We propose that in addition to the current assessments of music ability, additional aspects of music cognition should be examined in order to gain a fuller understanding of the links between music and speech processing. B.Z.’s performance on our novel tasks shows that an individual can be identified as having a congenital disorder of music processing, while also possessing the ability to appreciate aesthetic features of musical performance and to identify basic emotional colouring.

While further work will be required in the future in order to develop and standardise a range of stimuli and tasks to more extensively investigate the holistic prosodic qualities in question, we believe that a more holistic approach to the exploration of the auditory experience may be beneficial. While componential analysis of specific perceptual features in the processing of acoustic streams by employing artificial manipulations of stimuli has definitely enriched our perspective today, the study of individuals’ responses to naturally occurring prosodic cues can point to new promising directions.

## Funding

This work was supported by a Wellcome Trust-Birkbeck Institutional Strategic Support Fund [Project # ACR00.ACC01] to the first author.

## References

[b0005] Albouy P., Mattout J., Bouet R., Maby E., Sanchez G., Aguera P.E., Tillmann B. (2013). Impaired pitch perception and memory in congenital amusia: The deficit starts in the auditory cortex. Brain.

[b0010] Audibert N., Aubergé V., Rilliard A. (2005). The prosodic dimensions of emotion in speech: The relative weights of parameters. Interspeech.

[b0015] Ayotte J., Peretz I., Hyde K. (2002). Congenital amusia: A group study of adults afflicted with a music-specific disorder. Brain.

[b0020] Baird A.D., Walker D.G., Biggs V., Robinson G.A. (2014). Selective preservation of the beat in apperceptive music agnosia: A case study. Cortex.

[b0025] Besson M., Schön D., Moreno S., Santos A., Magne C. (2007). Influence of musical expertise and musical training on pitch processing in music and language. Restorative Neurology and Neuroscience.

[b0030] Bigand E., Poulin-Charronnat B. (2006). Are we “experienced listeners”? A review of the musical capacities that do not depend on formal musical training. Cognition.

[b0035] Bowen J.A. (1993). The history of remembered innovation: Tradition and its role in the relationship between musical works and their performances. The Journal of Musicology.

[b0040] Chen J.L., Kumar S., Williamson V.J., Scholz J., Griffiths T.D., Stewart L. (2015). Detection of the arcuate fasciculus in congenital amusia depends on the tractography algorithm. Frontiers in Psychology.

[b0045] Clynes M. (1995). Microstructural musical linguistics: Composers' pulses are liked most by the best musicians. Cognition.

[b0050] Crawford J.R., Garthwaite P.H. (2002). Investigation of the single case in neuropsychology: Confidence limits on the abnormality of test scores and test score differences. Neuropsychologia.

[b0055] Gosselin N., Paquette S., Peretz I. (2015). Sensitivity to musical emotions in congenital amusia. Cortex.

[b0060] Grossberg S., Pinna B. (2012). Neural dynamics of Gestalt principles of perceptual organization: From grouping to shape and meaning. Gestalt Theory.

[b0065] Hutchins S., Gosselin N., Peretz I. (2010). Identification of changes along a continuum of speech intonation is impaired in congenital amusia. Frontiers in Psychology.

[b0070] Hyde K.L., Lerch J.P., Zatorre R.J., Griffiths T.D., Evans A.C., Peretz I. (2007). Cortical thickness in congenital amusia: When less is better than more. The Journal of Neuroscience.

[b0075] Hyde K.L., Zatorre R.J., Griffiths T.D., Lerch J.P., Peretz I. (2006). Morphometry of the amusic brain: A two-site study. Brain.

[b0080] Hyde K.L., Zatorre R.J., Peretz I. (2011). Functional MRI evidence of an abnormal neural network for pitch processing in congenital amusia. Cerebral Cortex.

[b0085] Jiang C., Hamm J.P., Lim V.K., Kirk I.J., Yang Y. (2010). Processing melodic contour and speech intonation in congenital amusics with Mandarin Chinese. Neuropsychologia.

[b0090] Juslin P.N. (2013). From everyday emotions to aesthetic emotions: Towards a unified theory of musical emotions. Physics of Life Reviews.

[b0095] Kalmus H., Fry D.B. (1980). On tune deafness (dysmelodia): Frequency, development, genetics and musical background. Annals of Human Genetics.

[b0100] Liu F., Jiang C., Wang B., Xu Y., Patel A.D. (2015). A music perception disorder (congenital amusia) influences speech comprehension. Neuropsychologia.

[b0105] Liu F., Maggu A.R., Lau J.C., Wong P. (2015). Brainstem encoding of speech and musical stimuli in congenital amusia: Evidence from Cantonese speakers. Frontiers in Human Neuroscience.

[b0110] Liu F., Patel A.D., Fourcin A., Stewart L. (2010). Intonation processing in congenital amusia: Discrimination, identification and imitation. Brain.

[b0115] Lolli S.L., Lewenstein A.D., Basurto J., Winnik S., Loui P. (2015). Sound frequency affects speech emotion perception: Results from congenital amusia. Frontiers in Psychology.

[b0120] Loui P., Alsop D., Schlaug G. (2009). Tone deafness: A new disconnection syndrome?. The Journal of Neuroscience.

[b0125] Loutrari, A. and Lorch, M. (2018, in press). Music and language expressiveness: When emotional character does not suffice. In W. Thormaehlen, J. Kennaway, J. Prins and P. Gouk (eds.) *The Routledge Companion to Music, Mind and Wellbeing: Historical and Scientific Perspectives*. Routledge.

[b0130] Marin M.M., Thompson W.F., Gingras B., Stewart L. (2015). Affective evaluation of simultaneous tone combinations in congenital amusia. Neuropsychologia.

[b0135] Marques C., Moreno S., Castro S.L., Besson M. (2007). Musicians detect pitch violation in a foreign language better than nonmusicians: Behavioral and electrophysiological evidence. Journal of Cognitive Neuroscience.

[b0140] McDonald C., Stewart L. (2008). Uses and functions of music in congenital amusia. Music Perception: An Interdisciplinary Journal.

[b0145] Menninghaus W., Bohrn I.C., Knoop C.A., Kotz S.A., Schlotz W., Jacobs A.M. (2015). Rhetorical features facilitate prosodic processing while handicapping ease of semantic comprehension. Cognition.

[b0150] Omigie D., Müllensiefen D., Stewart L. (2012). The experience of music in congenital amusia. Music Perception: An Interdisciplinary Journal.

[b0155] Palmer C., Hutchins S. (2006). What is musical prosody?. Psychology of Learning and Motivation.

[b0160] Paraskevopoulos E., Tsapkini K., Peretz I. (2010). Cultural aspects of music perception: Validation of a Greek version of the Montreal Battery of Evaluation of Amusias. Journal of the International Neuropsychological Society.

[b0165] Patel A.D. (2003). Language, music, syntax and the brain. Nature Neuroscience.

[b0170] Patel A.D. (2008). Music, language, and the brain.

[b0175] Patel A.D., Foxton J.M., Griffiths T.D. (2005). Musically tone-deaf individuals have difficulty discriminating intonation contours extracted from speech. Brain and Cognition.

[b0180] Patel A.D., Wong M., Foxton J., Lochy A., Peretz I. (2008). Speech intonation perception deficits in musical tone deafness (congenital amusia). Music Perception: An Interdisciplinary Journal.

[b0185] Peretz I., Champod A.S., Hyde K. (2003). Varieties of musical disorders. Annals of the New York Academy of Sciences.

[b0190] Peretz I., Hyde K.L. (2003). What is specific to music processing? Insights from congenital amusia. Trends in Cognitive Sciences.

[b0195] Repp B.H. (1995). ‘Expressive Timing in Schumann’s “Träumerei”: An analysis of performances by graduate student pianists. Journal of the Acoustical Society of America.

[b0200] Schaal N.K., Pfeifer J., Krause V., Pollok B. (2015). From amusic to musical?—Improving pitch memory in congenital amusia with transcranial alternating current stimulation. Behavioural Brain Research.

[b0205] Schön D., Magne C., Besson M. (2004). The music of speech: Music training facilitates pitch processing in both music and language. Psychophysiology.

[b0210] Sloboda J.A. (1991). Music structure and emotional response. Psychology of Music.

[b0215] Stewart L. (2011). Characterizing congenital amusia. The Quarterly Journal of Experimental Psychology.

[b0220] Thompson W.F., Marin M.M., Stewart L. (2012). Reduced sensitivity to emotional prosody in congenital amusia rekindles the musical protolanguage hypothesis. Proceedings of the National Academy of Sciences.

[b0225] Tillmann B., Schulze K., Foxton J.M. (2009). Congenital amusia: A short-term memory deficit for non-verbal, but not verbal sounds. Brain and Cognition.

[b0230] Vuvan D.T., Nunes-Silva M., Peretz I. (2015). Meta-analytic evidence for the non-modularity of pitch processing in congenital amusia. Cortex.

[b0235] Wiethoff S., Wildgruber D., Kreifelts B., Becker H., Herbert C., Grodd W., Ethofer T. (2008). Cerebral processing of emotional prosody—Influence of acoustic parameters and arousal. Neuroimage.

[b0240] Zatorre R.J., Evans A.C., Meyer E. (1994). Neural mechanisms underlying melodic perception and memory for pitch. The Journal of Neuroscience.

